# Novel insights on [1,2]oxazolo[5,4‐*e*]isoindoles on multidrug resistant acute myeloid leukemia cell line

**DOI:** 10.1002/ddr.21962

**Published:** 2022-06-24

**Authors:** Manuela Labbozzetta, Marilia Barreca, Virginia Spanò, Maria Valeria Raimondi, Paola Poma, Monica Notarbartolo, Paola Barraja, Alessandra Montalbano

**Affiliations:** ^1^ Department of Biological, Chemical, and Pharmaceutical Sciences and Technologies (STEBICEF) University of Palermo Palermo Italy

**Keywords:** antimitotic agents, [1,2]oxazolo[5,4‐*e*]isoindoles, multidrug resistance

## Abstract

A series of [1,2]oxazolo[5,4‐*e*]isoindole derivatives was evaluated against HL‐60 cell line and its multidrug resistance (MDR) variant, HL‐60R, resistant to doxorubicin and to other P‐gp substrates by overexpressing the efflux pump. They displayed antiproliferative activities, with IC_50_ values ranging from 0.02 to 5.5 µM. In particular, the newly synthesized compound **4k** produced synergistic effects in terms of cell growth inhibition and cell death induction either in combination with a Vinca alkaloid, Vinblastine, and a Taxane, Paclitaxel in HL‐60R cells. The study of the mechanism of action indicated that all compounds showed antimitotic activity through inhibition of tubulin polymerization. Thus, [1,2]oxazoles could represent a valuable tool to overcome MDR mechanism, confirming the potential use of this class of compounds.

## 1 INTRODUCTION

Multidrug resistance (MDR), both innate and acquired, may be the cause of therapeutic failure in neoplastic diseases (Assaraf et al., 2019; Kaye, 1988; Szakács et al., 2006). The resistance can involve different classes of anticancer drugs, which possess different structures or mechanisms of action (Waghray & Zhang, 2018).

The mechanism leading to MDR is complex and frequently associated with different factors, such as enhanced DNA repair, inhibition of cancer cell apoptosis, alteration of signaling pathways, alterations in drug metabolism enzymes, drug sequestration in intracellular organelles and overexpression of efflux drug transporters (Dong et al., 2020; H. Zhang et al., 2021). The last can be considered the main mechanism of chemoresistance and is mediated by an overexpression of transmembrane efflux pumps (H. Zhang et al., 2021). They cause a decrease in intracellular drug concentration, which is responsible of the failure of the therapeutic treatment. The first identified and well‐characterized MDR transporter involved in drug efflux is the P‐glycoprotein (P‐gp), which is part of adenosine triphosphate (ATP) binding cassette (ABC) transporters, usually involved in different physiological functions as transporting lipids, sterols, peptides, toxins, and ions (Waghray & Zhang, 2018). P‐gp overexpression is related to decreased chemotherapeutic response both in blood cancers and solid tumors. In fact, some tumors, such as leukemia and breast cancer, which show low level of P‐gp expression at the beginning of treatment, can display its upregulation as a result of chemotherapy. Several important antitumor drugs are substrates of P‐gp, for example, the DNA‐intercalating anthracyclines (doxorubicin and daunorubicin), the topoisomerase inhibitors (topotecan and etoposide), and the tyrosine kinase inhibitors (Arribas et al., 2020; Cilibrasi et al., 2022) among which dasatinib and gefitinib (Waghray & Zhang, 2018).

Even in leukemic patients, the phenomenon of MDR is reputed to be responsible for a strong limitation of therapeutic choice, and for this reason it has been studied for years and continues to receive great attention (Du & Chen, 2017; Leith, 1998; Oliai & Schiller, 2020). For example, in acute myeloid leukemia (AML), drug resistance toward standard chemotherapeutic compounds, due to overexpression of P‐gp, leads to failure of the treatment or relapse. This P‐gp overexpression can result in highly aggressive AML clone, which needs more intensive treatment and invasive procedures (Kunadt et al., 2020; J. Zhang et al., 2019). MDR mechanisms produce resistance to microtubule‐targeting agents (Van Vuuren et al., 2015), such as Vinca alkaloids and Taxanes. The latter, although approved for some types of solid tumors, are also investigated for hematological neoplastic diseases (Chao et al., 2017; Hijiya et al., 2009; Philchenkov et al., 2015; Schnerch et al., 2013; Trendowski et al., 2015; Barreca, Stathis et al., 2020).

**Scheme 1:**
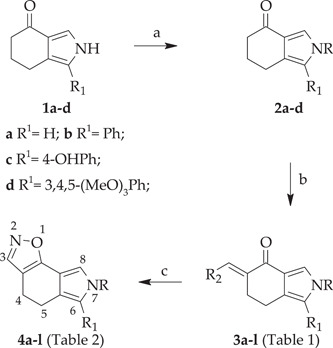
Synthetic strategy of substituted [1,2]oxazolo[5,4‐*e*]isoindoles **3a–3l** and **4a–4l**. (a) NaH, DMF, 0°C to rt, 1 h, then benzyl halide at 0°C to rt, 1–24 h, 60–88%; (b) *t*‐BuOK, toluene, 0°C to rt, 3 h then HCOOEt, rt, 24 h, 64%−87%; or TBDMAM, toluene, reflux, 24 h; (c) NH_2_OH·HCl, ethanol, reflux, 50 min, 57%−83%. DMF, dimethylformamide; TDBMAM, t‐butoxy‐bis‐(dimethylamino)‐methane.

In the course of our studies on small heterocyclic molecules with anticancer properties (Barreca, Spanò et al., 2022; Barreca, Ingarra et al., 2022), we discovered the class of the [1,2]oxazolo[5,4‐*e*]isoindole system of type **4** (Scheme 1), which showed antiproliferative activity against multiple tumor cell lines at micromolar—nanomolar level (Barreca et al., 2020; Spanò et al., 2020). [1,2]Oxazolo[5,4‐*e*]isoindoles were further investigated in two additional cell lines derived from human diffuse malignant peritoneal mesothelioma (DMPM), showing a dose‐dependent inhibition of cell proliferation in both cellular models, with IC_50_ values ranging from micromolar to nanomolar. The study of mechanism of action demonstrated the ability of [1,2]oxazole derivatives to impair cell cycle progression and induce apoptosis, as a consequence of the inhibition of tubulin polymerization. Selected derivatives, at well‐tolerated doses, were able to significantly reduce tumor volume in a DMPM xenograft model (Spanò, Pennati, Parrino, Carbone, Montalbano, Cilibrasi, et al., 2016; Spanò, Pennati, Parrino, Carbone, Montalbano, Lopergolo, et al., 2016).

On the basis of these observations, we wanted to examine the biological activity of these same compounds on AML cell line, HL‐60, and its MDR variant HL‐60R to assess their capacity to affect the MDR phenomenon. HL‐60R was obtained by us through exposure of the sensitive cell line HL‐60 with increasing doses of doxorubicin (Notarbartolo et al., 2002) and for this reason characterized by overexpression of P‐gp, constitutive activation of the transcription factor NF‐κB and as a consequence overexpression of inhibitor of apoptosis proteins (IAPs).

## 2 MATERIALS AND METHODS

### 2.1 Chemistry

All melting points were taken on a Büchi melting point M‐560 apparatus. IR spectra were determined in bromoform with a Shimadzu FT/IR 8400 S spectrophotometer. ^1^H and ^13^C NMR spectra were measured at 200 and 50 MHz, respectively, in DMSO‐*d*_6_ or CDCl_3_ solution using a Bruker Avance II series 200 MHz spectrometer. Column chromatography was performed with Merck silica gel (230–400 mesh ASTM) or a Büchi Sepacor chromatography module (prepacked cartridge system). Elemental analyses (C, H, N) were within ±0.4% of theoretical values and were performed with a VARIO EL III elemental analyzer.

Compounds **1a–1c, 2a–2j, 2l, 3a–3j, 3l, 4a–4j, 4l** were prepared according to our previously published procedures (Barraja et al., 2009; Spanò, Pennati, Parrino, Carbone, Montalbano, Cilibrasi, et al., 2016; Spanò, Pennati, Parrino, Carbone, Montalbano, Lopergolo, et al., 2016).

*Synthesis of 2‐(3,4‐dimethoxybenzyl)−1‐(3,4,5‐trimethoxyphenyl)−2,5,6,7‐tetrahydro‐isoindol‐4‐one (**2k**)*

To a solution of the suitable ketone **1c** (6 mmol) in anhydrous DMF (12 ml), NaH (6.6 mmol) was added at 0°C and the reaction was stirred for 1 h at room temperature. Then, 3,4‐dimethoxylbenzyl chloride (12 mmol) was added at 0°C, and the reaction mixture was stirred at room temperature for 16 h. Then the reaction mixture was poured onto ice and brine (40 ml), and the aqueous solution was extracted with dichloromethane (3 × 40 ml). The organic phase was dried over Na_2_SO_4_ and the solvent evaporated under reduced pressure. The crude product was purified by column chromatography (dichloromethane). Yellow oil; yield: 55%; IR: ν_max_ = 1687 (CO) cm^−1^; ^1^H NMR (200 MHz, CDCl_3_) *δ* 2.04–2.11 (m, 2H, CH_2_), 2.52 (t, *J* = 5.8 Hz, 2H, CH_2_), 2.66 (t, *J* = 5.8 Hz, 2H, CH_2_), 3.74 (s, 3H, CH_3_), 3.80 (s, 3H, CH_3_), 3.85 (s, 6H, 2 x CH_3_), 3.89 (s, 3H, CH_3_), 4.99 (s, 2H, CH_2_), 6.41 (s, 2H, H‐2' and H‐6'), 6.51–6.65 (2H, m, Ar), 6.74–6.82 (2H, m, Ar); ^13^C NMR (50 MHz, CDCl_3_) *δ* 22.4 (CH_2_), 23.7 (CH_2_), 37.0 (CH_2_), 54.9 (CH_2_), 56.7 (CH_3_), 56.8 (2 x CH_3_), 56.9 (CH_3_), 60.7 (CH_3_), 106.2 (2 x CH), 113.4 (CH), 113.5 (CH), 113.6 (C), 117.3 (C), 122.5 (CH), 128.5 (C), 130.0 (C), 131.5 (C), 136.4 (CH), 141.0 (C), 148.8 (C), 149.1 (C), 153.0 (2 x C), 189.6 (C). Anal. Calcd. for C_26_H_29_NO_6_: C 69.16, H 6.47, N 3.10. Found: C 69.33, H 6.61, N 2.95.

*Synthesis of 2‐(3,4‐dimethoxybenzyl)−5‐dimethylaminomethylene‐1‐(3,4,5‐trimethoxyphenyl)−2,5,6,7‐tetrahydroisoindol‐4‐one (**3k**)*

To a solution of ketone **2k** (5.3 mmol) in anhydrous toluene (10 ml), the Bredereck reagent *t*‐butoxy‐bis‐(dimethylamino)‐methane (TBDMAM) (3.31 ml, 16 mmol) was added under nitrogen atmosphere and the reaction mixture was heated under reflux for 24 h. After cooling, the solvent was evaporated at reduced pressure. The crude product was used in the next step without any further purification.

*Synthesis of 7‐(3,4‐dimethoxybenzyl)−6‐(3,4,5‐trimethoxyphenyl)−5,7‐dihydro‐4H‐[1,2]oxazolo[5,4‐e]isoindole (**4k**)*

To a solution of intermediate **3k** (5 mmol) in ethanol (15 ml), hydroxylamine hydrochloride (0.38 g, 5.5 mmol) was added and the reaction mixture was heated under reflux for 50 min. After cooling, the solvent was evaporated at reduced pressure. The crude product was purified by chromatography column using dichloromethane as eluent.

Yellow oil; yield 65%; ^1^H NMR (200 MHz, CDCl_3_) *δ* 2.71–2.82 (m, 4H, 2 x CH_2_), 3.73 (s, 6H, 2 x CH_3_), 3.79 (s, 3H, CH_3_), 3.85 (s, 3H, CH_3_), 3.89 (s, 3H, CH_3_), 5.01 (s, 2H, CH_2_), 6.44 (s, 2H, H‐2' and H‐6'), 6.55 – 6.68 (3H, m, Ar), 6.77–6.82 (1H, m, Ar), 7.06 (1H, s, Ar); ^13^C NMR (50 MHz, CDCl_3_) *δ* 23.0 (CH_2_), 24.0 (CH_2_), 50.9 (CH_2_), 55.8 (CH_3_), 55.9 (CH_3_), 56.0 (2 x CH_3_), 60.9 (CH_3_), 107.1 (2 x CH), 109.2 (C), 109.9 (CH), 110.6 (C), 111.3 (CH), 116.1 (CH), 119.0 (C), 119.1 (CH), 127.0 (C), 130.6 (C), 134.2 (C), 148.5 (C), 148.8 (CH), 149.1 (C), 153.1 (2 x C), 166.0 (C). Anal. Calcd. for C_27_H_28_N_2_O_6_: C 68.05, H 5.92, N 5.88. Found: C 67.91,H 6.08, N 5.97.

### 2.2 Pharmacological evaluation

#### 2.2.1 Cell lines and drugs

HL‐60 cells were obtained from ATCC® (CCL‐240), whereas its variant HL‐60R was selected for MDR by exposure to gradually increasing concentrations of doxorubicin. MCF‐7 cells were obtained from ATCC® (HTB‐22). The nontumorigenic cell lines hTERT‐RPE‐1 (ATCC, CRL‐4010) and 1‐7HB2 (ECACC 10081201‐Cancer Research Technology) was kindly provided by Prof. Patrizia Cancemi and Prof. Giulio Ghersi (STEBICEF Department, University of Palermo, Italy), respectively. Cells were cultured in a humidified atmosphere at 37°C in 5% CO_2_. After obtaining the cells, the first passage carried out was assigned passage number 1. Cells with a narrow range of passage number (4 ± 6) were routinely tested for Mycoplasma contamination and were used for all experiments. HL‐60 and HL‐60R cells were cultured in Roswell Park Memorial Institute (RPMI) 1640, while MCF‐7, hTERT‐RPE‐1, and 1‐7HB‐2 cells were cultured in Dulbecco's Modified Eagle Medium (DMEM) (HyClone Europe Ltd.) low glucose, in particular only for 1‐7HB‐2 cells supplemented with hydrocortisone (5 μg/ml) and insulin (10 μg/ml). All media were supplemented with 10% heat‐inactivated fetal calf serum, 2 mM l‐glutamine, 100 U/ml penicillin, and 100 μg/ml streptomycin (all reagents were from EuroClone S.p.A.; GE Healthcare Life Sciences).

Vinblastine and Paclitaxel were purchased from Sigma (Sigma‐Aldrich Srl), and all derivatives were dissolved in DMSO.

#### 2.2.2 Cell growth assays

Cells were seeded at 5 × 10^3^ cells/well onto 96 well plates and incubated overnight at 37°C. At time 0, the medium was replaced with fresh complete medium supplemented with the [1,2]oxazolo[5,4‐*e*]isoindoles at large concentrations range. Following 72 h of treatment, 15 µl commercial solution obtained from Promega Corp.  containing 3‐(4,5‐dimethylthiazol‐2‐yl)−5‐(3‐carboxymethoxyphenyl)−2‐(4‐sulphophenyl)−2H‐tetrazolium (MTS) and phenazine ethosulfate was added. The plates were incubated in a humidified atmosphere at 37°C in 5% CO_2_ for 2 h, and the bioreduction of MTS dye was evaluated by measuring the absorbance of each well at 490 nm using a microplate absorbance reader (iMark Microplate Reader; Bio‐Rad Laboratories, Inc.). Cell growth inhibition was expressed as a percentage of the absorbance of the control cells. Data were expressed as mean ± standard error (SE) of at least three different experiments performed in triplicate.

Potentiation or synergism due to cotreatments were evaluated by viable cell count with Trypan blue exclusion test. Data were expressed as mean ± SE of at least three different experiments performed in duplicate.

#### 2.2.3 Evaluation of cell death by flow cytometry

Cells were collected and washed twice with ice‐cold PBS and then resuspended in a hypotonic fluorochrome solution containing propidium iodide (PI) 50 µg/ml in 0.1% sodium citrate plus 0.03% (v/v) Nonidet P‐40, at 1 × 10^6^/ml. After incubation (at least 1 h at 4°C) in this solution, the samples were filtered through nylon cloth, 40 µm mesh, and their fluorescence was analyzed using a FACSCanto instrument (Becton Dickinson). The data were analyzed with BD FACSDiva software v.6.1.2. (Becton Dickinson). Cell death was determined by evaluating the percentage of events accumulated in the preG_0_‐G_1_ position.

#### 2.2.4 Western blot analysis

Whole‐cell lysates were obtained from HL‐60R cells using RIPA buffer (Santa Cruz Biotechnology Inc.) and 25 µg protein was subjected to 10% SDS‐PAGE and transferred to nitrocellulose membrane (Amersham, Pharmacia Biotech) using a semi‐dry fast blot apparatus (Bio‐Rad). Membranes were blocked with 5% (w/v) BSA in PBS‐0.1% (v/v) Tween 20 for 1 h and then filters were incubated with primary antibodies raised against GAPDH (1:20,000; Sigma‐Aldrich Srl Milan), XIAP (1:500; Cell Signaling Technology, Inc), survivin (1:2000; Abcam Limited), Bcl‐2 (1:1000; Santa Cruz Biotechnology Inc.) and IAP‐1 (1:1000; Cell Signaling Technology, Inc). Hybridization was visualized using an enhanced chemiluminescence detection kit (SuperSignal West Femto Maximum Sensitivity Substrate, Thermo Scientific Life Technologies Italia) and the Versa DOC imaging system (BioRad Laboratories). Immunoblots were quantified by densitometry measurements after normalization with glyceraldehyde‐3‐phosphate dehydrogenase and results were expressed as fold changes.

#### 2.2.5 Determination of doxorubicin accumulation

The effects of **4e** and **4k** on intracellular accumulation of doxorubicin were evaluated in the HL‐60R cell line. The cells were seeded in 24‐well plates at a density of 100,000/well cells and after 24 h, the cells were treated with compounds, at their IC_50_ or with verapamil 10 µM. After 24 h of incubation doxorubicin 2 µM was added for 1 h. The cells were subsequently washed with PBS twice and then resuspended in a final volume of 400 µl of PBS. The fluorescence intensity of doxorubicin was measured by flow cytometry using a FACSAria III instrument (Becton Dickinson). The results are reported as a percentage of the fluorescence intensity relative to the control (mean± SE of three experiments).

#### 2.2.6 P‐gp ATPase activity determination

P‐gp ATPase activity was performed with Pgp‐Glo™ Assay Systems (Promega) following manufacturer's instructions. The compounds **4e** and **4k**, at their IC_50s_ (test compound [TC]) were added to a 96‐well white plate in duplicate and incubated with recombinant human P‐gp membranes. The compound Na_3_VO_4_ (0.25 mM) was used as the selective inhibitor of P‐gp ATPase activity while negative control (no treatment [NT]) with only Pgp‐GLO assay buffer was used to provide a measure of unregulated ATPase activity. Verapamil (0.5 mM) is a P‐gp substrate that stimulates P‐gp ATPase activity and represents the positive control for drug stimulation of P‐gp ATPase activity. In fact, it is not a pure inhibitor of P‐gp function as well as Na_3_VO_4_, but in association with another P‐gp substrate like doxorubicin, it behaves as a competitive antagonist, that is, an efflux inhibitor. The ATP Standards curve serves to verify the correct execution of the assay. MgATP (5 mM) was added to initiate the ATPase activity; after 40 min incubation at 37°C, the reaction was stopped with 50 µl ATPase Detection Reagent and then incubated for 20 min at room temperature.

Luminescence was measured using a GLOMAX Multidetection System (Promega). The data were presented as change in luminescence (ΔRLU), calculated as follows: ΔRLU_basal_ is the difference between the average luminescent signals from Na_3_VO_4_‐treated samples (RLU_Na3VO4_) and untreated (NT) samples (RLU_NT_), ΔRLU_TC_ that reflects P‐gp ATPase activity in the presence of the test compounds, is the difference between the average luminescent signals from Na_3_VO_4_‐treated samples (RLU_Na3VO4_) and test compound‐treated samples (RLU_TC_).

#### 2.2.7 Tubulin polymerization assays

HL‐60 and HL‐60R cells were seeded in 6‐well plates and after 24 h were treated with Vinblastine (VIN), Paclitaxel (PTX), and compound **4k** for 24 h at concentrations corresponding to the IC_50_ at 72 h. To separate cytosolic and cytoskeletal‐associated proteins, cells were rinsed in PEM buffer (0.1 M PIPES, pH 6.94; 2 mM EGTA; 1 mM MgCl_2_; 10% glycerol; 1 µM protease inhibitors cocktail; 0.5% Nonidet NP‐40), lysed at room temperature for 10 min with the same buffer (200 µl). After centrifugation (14,000 rpm at 4°C for 10 min), the soluble fraction (supernatant) was separated from polymerized fraction (pellet); the latter was resuspended in the same buffer (200 µl).

Both the fractions were then diluted 3:1 with 4 × SDS‐PAGE sample buffer. Proteins were separated by SDS‐PAGE, and tubulin distribution was analyzed by immunoblotting using anti‐α‐tubulin antibody (Sigma‐Aldrich). Hybridization was visualized using an enhanced chemiluminescence detection kit (SuperSignal West Femto Maximum Sensitivity Substrate, Thermo Scientific Life Technologies Italia) and the Versa DOC imaging system (BioRad Laboratories).

### 2.3 Statistical analysis

Results are given as means ± SE. Statistical analysis was carried out by analysis of variance (one‐way analysis of variance) followed by Tukey's test. Statistica ver. 12 (StatSoft Inc. 1984–2014) was used as software for the analyses.

## 3 RESULTS AND DISCUSSION

### 3.1 Chemistry

[1,2]Oxazolo[5,4‐*e*]isoindoles of type **4** were prepared following the synthetic route depicted in Scheme 1. Tetrahydroisoindole‐4‐one **1a–1d** were obtained according to our published procedures (Barraja et al., 2009; Spanò, Pennati, Parrino, Carbone, Montalbano, Cilibrasi, et al., 2016; Spanò, Pennati, Parrino, Carbone, Montalbano, Lopergolo, et al., 2016). Ketones **1** were functionalized at the pyrrole nitrogen using different benzyl halides, sodium hydride as base, in *N,N*‐dimethylformamide (DMF), allowing the isolation of ketones of type **2** (60%–88%) (Scheme 1). These latter were converted into key intermediates **3a–3g, 3i, 3j** by treatment with ethyl formate, in presence of potassium *tert*‐butoxyde (*t*‐BuOK) as base (64%–87%) (Scheme 1, Table 1). Alternatively ketones **2** were reacted with *tert*‐butoxy bis(dimethylamino)methane (TBDMAM) in refluxing toluene to give enaminoketone intermediates **3h, 3k, 3l** which were not isolated as pure compounds, and thus used as crude products directly in the next step (Scheme 1, Table 1).

**Table 1:** Overview of α‐substituted ketones **3a**–3**l**

CPD	Substrate	R	R^1^	R^2^
**3a**	**2a**	Bn	H	OH
**3b**	**2b**	2‐MeOBn	H	OH
**3c**	**2c**	3‐MeOBn	H	OH
**3d**	**2d**	3,4‐(MeO)_2_Bn	H	OH
**3e**	**2e**	3,4,5‐(MeO)_3_Bn	H	OH
**3f**	**2f**	4‐MeOBn	Ph	OH
**3g**	**2g**	3,4‐(MeO)_2_Bn	Ph	OH
**3h**	**2h**	3‐NO_2_,4‐MeOBn	Ph	NMe_2_
**3i**	**2i**	4‐MeOBn	4‐OHPh	OH
**3j**	**2j**	4‐MeOBn	3,4,5‐(MeO)_3_Ph	OH
**3k**	**2k**	3,4‐(MeO)_2_Bn	3,4,5‐(MeO)_3_Ph	NMe_2_
**3l**	**2l**	3‐NO_2_,4‐MeOBn	3,4,5‐(MeO)_3_Ph	NMe_2_

Both key intermediates **3** are highly reactive toward dinucleophiles. Thus, ring closure leading to [1,2]oxazoles was achieved by reaction of intermediates **3** with hydroxylamine hydrochloride in refluxing ethanol to give the desired tricyclic derivatives **4** in good yield (57%−83%) (Scheme 1, Table 2).

**Table 2:** Overview of α‐substituted ketones **4a–4l**

CPD	R	R^1^	CPD	R	R^1^
**4a**	Bn	H	**4g**	3,4‐(MeO)_2_Bn	Ph
**4b**	2‐MeOBn	H	**4h**	3‐NO_2_,4‐MeOBn	Ph
**4c**	3‐MeOBn	H	**4i**	4‐MeOBn	4‐OHPh
**4d**	3,4‐(MeO)_2_Bn	H	**4j**	4‐MeOBn	3,4,5‐(MeO)_3_Ph
**4e**	3,4,5‐(MeO)_3_Bn	H	**4k**	3,4‐(MeO)_2_Bn	3,4,5‐(MeO)_3_Ph
**4f**	4‐MeOBn	Ph	**4l**	3‐NO_2_,4‐MeOBn	3,4,5‐(MeO)_3_Ph

### 3.2 Biology

#### 3.2.1 Anticancer activity

[1,2]Oxazolo[5,4‐*e*]isoindoles were tested to evaluate their cytotoxic activity on HL‐60 cells and its MDR variant HL‐60R, by MTS assay. In addition, to evaluate the antiproliferative activity on a different tumor model, we have chosen a breast cancer cell line, MCF‐7, and to confirm the absence of toxicity of the compounds on non‐tumorigenic models, we have selected two different non‐tumorigenic cell lines, hTERT‐RPE‐1 and 1‐7HB‐2, (Table 3). Interestingly, it was observed that differently from the reference anti‐mitotic drugs, all derivatives induced cell growth inhibition at similar concentration in both tumor cell lines. Notably, nontumorigenic cells are not affected by [1,2]oxazoles at their maximum concentration (μM) used in the MTS assay. The best results were obtained for derivative **4e**, belonging to the 1,3‐H substituted series and bearing a 3,4,5‐trimethoxybenzyl group at the pyrrole nitrogen, which maintained nanomolar antiproliferative activity against both HL‐60 and HL‐60R cell lines. Moreover, **4c** and **4d** belonging to the same series, bearing a 3‐methoxybenzyl and a 3,4‐dimethoxybenzyl group on the pyrrole nitrogen respectively, were the second best showing antiproliferative activity at submicromolar level. Moving the methoxy group from position 3 to position 2 in the benzyl moiety (**4b**) or its removal (**4a**) generated a decrease of the activity but still at low micromolar level. Among [1,2]oxazoles bearing differently substituted phenyl substituents at position 6 of the tricyclic core system, the best activity was obtained for derivatives **4f**, **4i**, and **4j**, bearing a 4‐methoxybenzyl group at the pyrrole nitrogen, which showed comparable activity at submicromolar level. The introduction of a nitro group in position 3 of the benzyl moiety for the 6‐phenyl derivative led to compound **4h**, which maintains the activity in the submicromolar range (compare with **4f**). Compound **4g** of the same series showed micromolar activity. Decoration of the tricyclic system with a 3,4,5‐trimethoxyphenyl group at position 6 led to a decrease of the activity at low micromolar level when the R group is a 3‐nitro,4‐methoxybenzyl (**4l**) and a 3,4‐dimethoxybenzyl (**4k**). The only exception is for **4j** (R = 4‐MeOBn) showing potent activity at submicromolar level, indicating that a double substitution at the benzyl moiety in this series is not tolerated.

**Table 3:** IC_50_ values of [1,2]oxazolo[5,4‐*e*]isoindoles **4a–4l** in HL‐60 and HL‐60R cells (μM)

CPD	HL‐60 (IC_50_ ± SE)	HL‐60R (IC_50_ ± SE)	MCF‐7 (IC_50_)	h‐TERT‐RPE‐1 (IC_50_)	1‐7HB2 (IC_50_)
**4a**	1.90 ± 0.90	2.10 ± 0.20	>50.0	>20.0	>20.0
**4b**	2.10 ± 0.40	2.40 ± 0.80	>50.0	>20.0	>20.0
**4c**	0.25 ± 0.10	0.30 ± 0.07	>50.0	>5.0	>5.0
**4d**	0.60 ± 0.01	0.70 ± 0.09	>50.0	>5.0	>5.0
**4e**	0.02 ± 4.00	0.04 ± 7.00	>5.0	>0.10	>0.10
**4f**	0.30 ± 0.00	0.20 ± 8.00	>50.0	>5.0	>5.0
**4g**	1.40 ± 0.45	2.90 ± 0.70	>50.0	>20.0	>20.0
**4h**	0.25 ± 0.20	0.27 ± 0.25	>50.0	>5.0	>5.0
**4i**	0.27 ± 0.03	0.30 ± 0.03	>50.0	> 5.0	>5.0
**4j**	0.20 ± 0.04	0.26 ± 0.04	>50.0	>5.0	>5.0
**4k**	3.00 ± 0.25	5.50 ± 1.40	>50.0	>20.0	>20.0
**4l**	2.25 ± 1.00	2.30 ± 0.75	>50.0	>20.0	>20.0
Vinblastine	0.05 ± 0.90	0.82 ± 0.02	‐	‐	‐
paclitaxel	0.02 ± 0.04	1.30 ± 0.14	‐	‐	‐

*Note*: For MCF‐7, hTERT‐RPE‐1 and 1‐7HB2 cell lines, the maximum concentration (μM) used in the MTS assay is reported. Data were expressed as mean ± SE of at least three different experiments performed in triplicate.

The good results obtained against the HL‐60R cell line led us to hypothesize that the [1,2]oxazolo isoindoles **4a–4l** are able to overcome MDR, at least the one induced by the overexpression of P‐gp, differently from VIN and PTX, respectively belonging to Vinca alkaloids and Taxanes, used in our study.

In fact, in both cases (for VIN and PTX), the antiproliferative activity against the HL‐60R showed a decrease up to two orders of magnitude with respect to the HL‐60 cell line. On the contrary, this difference is missing in all [1,2]oxazolo isoindoles **4a–4l** tested, showing equal potency against the two cell lines. Thus, we further studied the effect in combination with Verapamil, a P‐gp inhibitor. In this case, we did not observe any potentiation or synergistic effects, confirming that the derivatives might not be substrates of the P‐gp efflux pump.

Results obtained on a breast cancer cell line, MCF‐7, in which the IC_50_ was not achieved at concentrations far above the range used for AML cells, lead us to conclude that all compounds probably have a tumor‐specific action; myeloid leukemia cells are in fact much more sensitive and responsive to their antiproliferative action.

Finally, we analyzed the effects of all derivatives and VIN or PTX alone or in combination in the inhibition of cell growth by viable cell count with Trypan blue exclusion test, at sub‐cytotoxic concentrations. Table 4 shows the percentages of cell growth inhibition obtained by co‐treatment versus percentages expected. It can be observed that only compounds **4a**, **4c**, **4l**, and **4k** showed a synergistic effect in the HL‐60R cell line. Surprisingly, different behaviors were observed: compounds **4a**, **4c**, and **4l** synergized only with PTX (1 μM) with a range of percentage of cell growth inhibition equals to 54.5%–81.0%. On the contrary, compound **4k** was the only one which produced a potentiation effect in the MDR cell line in combination both with VIN (60.0%) and PTX (80.5%).

**Table 4:** Cell counting analysis in HL‐60R cells following 48 h of treatment with **4a**, **4c**, **4k**, and **4l**, **PTX** (1 µM) and **VIN** (0.3 µM), either alone or in combination

Treatments	Cell growth inhibition (%)	Expected (%)
Paclitaxel (PTX)	13.0 ± 9.2	
**4a** (1 µM)	2.5 ± 1.8	
**4a** (1 µM) + PTX	81.0 ± 3.5	15.5 ± 7.4*
**4c** (0.2 µM)	42.5 ± 0.3	
**4c** (0.2 µM) + PTX	65.0 ± 9.2	50.0 ± 5.6
**4k** (1.5 µM)	16.0 ± 5.7	
**4k** (1.5 µM) + PTX	80.5 ± 0.3	28.0 ± 2.8**
**4l** (1 µM)	0.0 ± 0.0	
**4l** (1 µM) + PTX	54.5 ± 5.3	13.0 ± 9.2
VIN	0.0 ± 0.0	
**4k** (1.5 µM) + VIN	60.0 ± 5.0	16.0 ± 5.7*

*Note*: *p < 0.05, **p < 0.01 versus observed. Data are expressed as the mean ± standard error of three independent experiments. Expected value: multiplication of the effects of the agents alone (Aapro et al., 1983; Labbozzetta et al., 2009).

Even though derivative **4e** was the most potent within the series, it was not detected any potentiation with the reference drugs.

**Figure 1:**
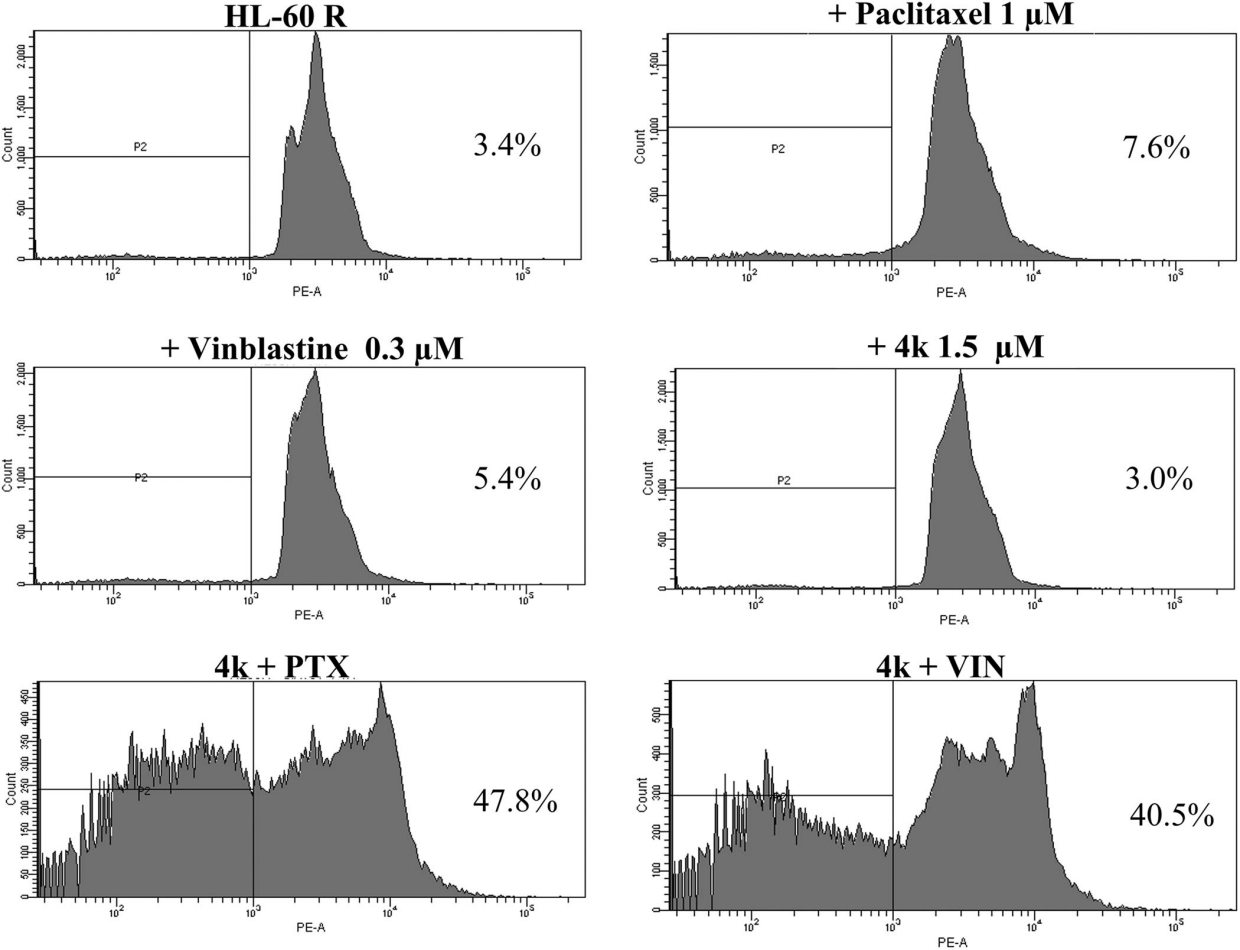
Induction of cell death by **4k**, Paclitaxel (PTX), and Vinblastine (VIN). Representative example of flow cytometry analysis in HL‐60R cells treated for 48 h with **4k**, PTX, and VIN at different concentrations, either alone or in combination. Profiles of propidium iodide‐stained DNA are shown. Percentages of the events accumulated in the pre G_0_‐G_1_ position in each panel are indicated.

Although **4k** is not the most powerful compound within the series in terms of antiproliferative activity, it is the only one showing a potentiation of the effect in combination with classic antimitotics. This result is also confirmed by cell death evaluation by flow cytometry analysis of DNA stained with propidium iodide. In fact, compound **4k** caused a strong synergistic effect in induction of cell death both in combination with PTX and VIN in HL‐60R cells (Figure 1, Table 4).

**Figure 2:**
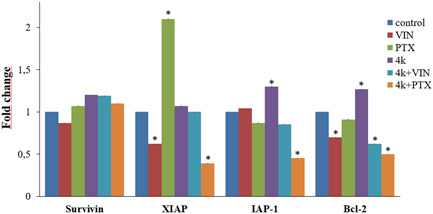
Western blot analysis of the levels of survivin, X‐linked inhibitor of apoptosis protein (XIAP), IAP‐1, and Bcl‐2 in HL‐60R cells. Quantitative estimations of the protein levels were determined by densitometry measurements after normalization with glyceraldehyde‐3‐phosphate dehydrogenase. The results expressed as mean ± standard error of three different experiments. Differences when treatments are compared to the control: *p < .01 (Tukey test).

IAPs are able to block apoptosis induced by different triggers including antitumor agents. For this reason, the overexpression of antiapoptotic proteins such as Bcl‐2, or IAPs, for example, survivin, XIAP (X‐Linked IAPs), IAP‐1, is responsible for multi‐drug resistance. The last result was corroborated by a substantial protein expression decrease of some antiapoptotic factors such as, XIAP, IAP‐1, and Bcl‐2, enhanced by the co‐treatment of **4k** and PTX (Figure 2). In addition, we observed in some cases a significant increase of protein expression when HL‐60R cells were treated with PTX or **4k** alone. As reported by other authors, PTX, in fact, can cause an activation of NF‐κB signaling and consequently an increase of its target such as XIAP (Aggarwal et al., 2005). For this reason, the results obtained highlight the importance of the synergic effects of cotreatments.

#### 3.2.2 Effects of compounds on P‐gp activity

**Figure 3:**
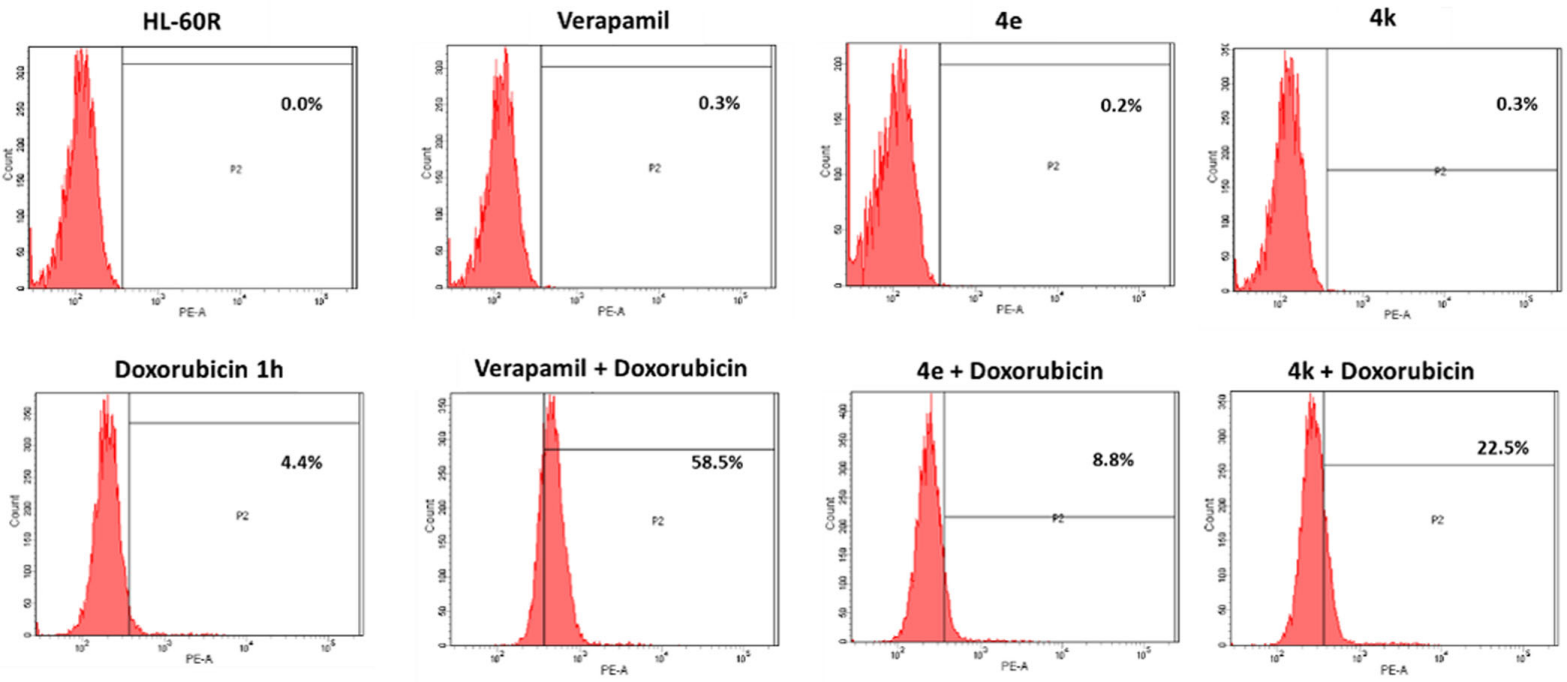
Effects of compounds on intracellular accumulation of doxorubicin in HL‐60R cell line. Representative example of flow cytometry analysis of intracellular accumulation of doxorubicin (2 µM) after 1 h of incubation in HL‐60R cells pretreated with **4e** 0.04 µM, **4k** 5.50 µM or verapamil 10 µM for 24 h (Labbozzetta et al., 2022; Molinari et al., 1998).

Since different behaviors were observed when [1,2]oxazolo isoindoles **4a–4l** were tested in combination with PTX and VIN, we decided to further investigate if these differences could be due to specific alterations of P‐gp function. Thus, it was investigated the ability of selected derivatives (**4e** and **4k**) to modulate the intracellular accumulation of doxorubicin, a fluorescent substrate of P‐gp, in HL‐60R cells. In particular, derivative **4e** was selected since it did not display any synergistic effects in combination with P‐gp substrates, despite being the most active compound within the series. On the contrary, **4k** was able to synergize with PTX and VIN both in terms of cellular proliferation inhibition and induction of cell death. HL‐60R cells were treated with **4e** and **4k** at their IC_50s_ for 24 h, while verapamil (10 µM) was used as positive control. After incubation, doxorubicin (2 µM) was added, and its intracellular accumulation was measured by flow cytometric analysis after the proper time (1 h). The results, reported as percentage of fluorescence intensity (Figure 3, Table 5), proved a low increase in fluorescence in HL‐60R cells pretreated with **4e** and a more evident one in the case of compound **4k**.

**Table 5:** Effects of compounds on intracellular accumulation of doxorubicin in HL‐60R cell lines

	Fluorescence (%)
Treatment	HL‐60R
Control	0.0 ± 0.0
Verapamil 10 µM	0.3 ± 0.1
**4e** 0.04 µM	0.2 ± 0.07
**4k** 5.50 µM	0.3 ± 0.1
Doxo 2 µM 1 h	4.4 ± 0.3
Verapamil 10 µM + Doxo 2 µM 1 h	58.5 ± 2.5**
**4e** 0.04 µM + Doxo 2 µM 1 h	8.8 ± 0.3*
**4k** 5.50 µM + Doxo 2 µM 1 h	22.5 ± 1.1**

*Note*: *p < .05, **p < .01 are significant differences among the treatments in combination compared to doxorubicin alone (one‐way analysis of variance followed by Tukey's test). HL‐60R cells were treated with **4e** 0.04 µM, **4k** 5.50 µM, or verapamil 10 µM. After 24 h of incubation, doxorubicin 2 µM was added. The intracellular accumulation of doxorubicin was measured after 1 h by flow cytometric analysis. The results are reported as percentage of fluorescence intensity (means ± standard error of three experiments).

**Figure 4:**
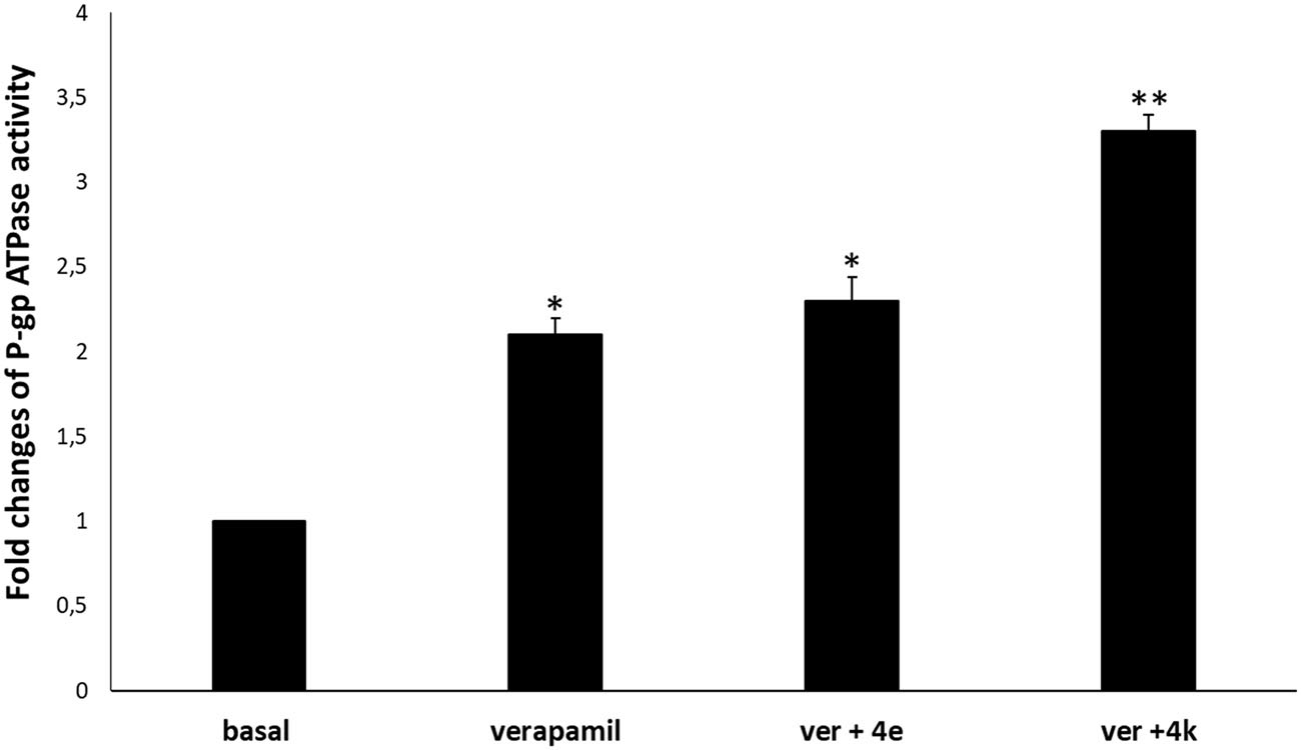
Effects of compounds **4e** and **4k** (at their IC_50s_) on verapamil‐stimulated P‐gp ATPase activity. The data are expressed as fold changes of P‐gp ATPase activity compared to basal one (ΔRLU_TC_/ΔRLU_basal_) and are presented as mean ± SE of three experiments, each in duplicate. Differences when treatments are compared to the basal activity, *p ˂ .05, **p < .01 (one‐way analysis of variance followed by Tukey's test).

To have a deeper insight into the mechanism of action of our compounds, effects of compounds **4e** and **4k** on P‐gp ATPase activity were also evaluated by the P‐gp‐Glo™ assay, which detects a luminescent signal inversely proportional to the ATP consumption. Verapamil was used as positive control, being a substrate for transport by P‐gp and stimulator of ATP‐dependent drug efflux transporter. Only compound **4k** produced a significative increase of verapamil‐stimulated P‐gp ATPase activity (Figure 4).

#### 3.2.3 Antimitotic activity

**Figure 5:**
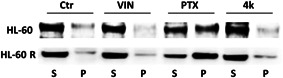
Representative western blot showing the soluble (S) or polymerized (P) tubulin fraction in HL‐60 and HL‐60R cells. Cells were treated for 24 h with vinblastine (VIN), PTX, and **4k** at the concentrations corresponding to the IC_50_ at 72 h. Vinblastine and Paclitaxel were used as reference drugs due to their opposite mechanism of action on tubulin polymerization. The experiment repeated three times gave the same result.

On the basis of previous evidence (Spanò, Pennati, Parrino, Carbone, Montalbano, Cilibrasi, et al., 2016; Spanò, Pennati, Parrino, Carbone, Montalbano, Lopergolo, et al., 2016) of new analogs, we performed a Western blot analysis analysis to verify the antimitotic activity of compound **4k**. Although both our cell lines are characterized by a very low polymerized fraction of tubulin, the result provided by compound **4k** clearly indicates a similar behavior to that of Vinca alkaloid, increasing the soluble fraction of tubulin in both the HL‐60 and HL‐60R cell lines (Figure 5) differently from taxol, which instead determines a marked increase in the polymerized fraction compared to the untreated control, thus confirming the role of **4k** as an antimitotic agent and its mechanism.

## 4 CONCLUSIONS

In this study, a series of [1,2]oxazolo[5,4‐*e*]isoindole derivatives were synthesized and evaluated against HL‐60 and its MDR variant HL‐60R cell lines. All tested compounds displayed inhibitory activities, with IC_50_ values ranging from 0.02 to 5.50 µM. The majority of compounds (7 out 12), namely **4c**, **4d**, **4e**, **4f**, **4h**, **4i**, and **4j** (IC_50_ < 1 µM) were equally active against both cell lines suggesting their ability to overcome the MDR, depending by P‐gp overexpression.

The new [1,2]oxazole **4k**, in particular, produced an important potentiation effect in terms of cell growth inhibition either when tested in combination with VIN and PTX in HL‐60R cells. Overall, **4k** seems to have a similar behavior to verapamil on P‐gp function. It could act, in fact, as competitive modulator of the efflux pump causing intracellular accumulation of the P‐gp substrate (doxorubicin) in a MDR cell line. HL‐60R cell line is resistant to doxorubicin and to other P‐gp substrates as antimitotic drugs, by overexpressing the efflux pump or, hyper‐activation of NF‐κB and overexpression of its target as antiapoptotic factors (Notarbartolo et al., 2002, 2004; Sikic et al., 1997; Vanhoefer et al., 1996; Wang et al., 2016). Thus, the strong synergism of action of **4k** in combination both with VIN and PTX in the induction of cell death caused by the hyperactivation of IAPs, appears to be a significant result. Insight on the mechanism of action confirmed the antimitotic activity of **4k** through inhibition of tubulin polymerization.

The potent antiproliferative effect confirmed against HL‐60R cell line demonstrates that derivatives **4a–4l** could further be explored as valuable tool to overcome MDR mechanism thus confirming the potentialities of the class of compounds and encouraging to conduct further studies.

## AUTHOR CONTRIBUTIONS

Marilia Barreca and Virginia Spanò carried out synthesis and characterizations; Manuela Labbozzetta performed biological studies; Maria V. Raimondi and Paola Poma supervision and draft preparation; Monica Notarbartolo, Alessandra Montalbano and Paola Barraja supervision and writing—original draft preparation. All authors have read and agreed to the published version of the manuscript.

### 2 CONFLICT OF INTEREST

The authors declare no conflict of interest.

### 3 DATA AVAILABILITY STATEMENT

The data that support the findings of this study are openly available in IRIS UniPA (https://iris.unipa.it/).
